# Lightweight Optic Disc and Optic Cup Segmentation Based on MobileNetv3 Convolutional Neural Network

**DOI:** 10.3390/biomimetics9100637

**Published:** 2024-10-18

**Authors:** Yuanqiong Chen, Zhijie Liu, Yujia Meng, Jianfeng Li

**Affiliations:** 1 School of Computer Science and Engineering, Central South University, Changsha 410000, China; yqchen@vip.163.com; 2 School of Computer Science and Engineering, Jishou University, Zhangjiajie 427000, China

**Keywords:** glaucoma screening, optic disc and optic cup segmentation, convolutional neural network, adversarial generative network

## Abstract

Glaucoma represents a significant global contributor to blindness. Accurately segmenting the optic disc (OD) and optic cup (OC) to obtain precise CDR is essential for effective screening. However, existing convolutional neural network (CNN)-based segmentation techniques are often limited by high computational demands and long inference times. This paper proposes an efficient end-to-end method for OD and OC segmentation, utilizing the lightweight MobileNetv3 network as the core feature-extraction module. Our approach combines boundary branches with adversarial learning, to achieve multi-label segmentation of the OD and OC. We validated our proposed approach across three public available datasets: Drishti-GS, RIM-ONE-r3, and REFUGE. The outcomes reveal that the Dice coefficients for the segmentation of OD and OC within these datasets are 0.974/0.900, 0.966/0.875, and 0.962/0.880, respectively. Additionally, our method substantially lowers computational complexity and inference time, thereby enabling efficient and precise segmentation of the optic disc and optic cup.

## 1. Introduction

Glaucoma is a chronic eye condition and ranks as the second-most prevalent cause of blindness globally. Experts predict that glaucoma patients worldwide will increase from 64.3 million in 2013 to 118 million in 2040 [1,2]. At present, there are three main methods for glaucoma diagnosis based on information technology: The first method involves measuring intraocular pressure (IOP), which tends to increase in glaucoma patients, due to an imbalance between intraocular fluid production and drainage. However, a significant number of glaucoma patients exhibit minimal changes in IOP, leading to reduced diagnostic accuracy using this approach. The second method employs visual field (VF) evaluation, which necessitates sophisticated medical equipment, and involves subjective diagnosis steps. Although widely used, this method presents challenges, in terms of equipment requirements and subjective interpretation. The third method, image evaluation of the optic nerve head (ONH), predominantly relies on the analysis of digital fundus images (DFI) for glaucoma diagnosis and is commonly employed in clinical practice. However, these existing diagnostic methods are characterized by high costs and low efficiency, rendering them unsuitable for large-scale glaucoma screening and diagnosis. Due to the swift advancement of information technology, auxiliary diagnostic technology is crucial for large-scale glaucoma diagnosis and screening. Excellent auxiliary diagnosis methods can significantly reduce the cost of diagnosis while improving the accuracy of clinical diagnosis. Among these auxiliary diagnostic techniques, the vertical cup-to-disc ratio (CDR) serves as a widely accepted reference standard. Accurate segmentation of the optic disc (OD) and optic cup (OC) is essential for precise CDR measurement and forms the basis for reliable auxiliary glaucoma diagnosis.

Recently, deep learning techniques have shown significant progress in the segmentation of medical images. Compared to traditional methods [3], deep learning-based segmentation approaches generally outperform conventional methods, in terms of accuracy and efficiency. Numerous researchers have made notable advancements in this field. For instance, reference [4] introduced a general encoder–decoder network for segmenting the optic disc (OD) and optic cup (OC), which includes a multi-scale weight-shared attention (MSA) module and a densely connected depthwise separable convolution (DSC) module. Additionally, reference [5] introduced an unsupervised domain adaptation framework—namely, input and output space unsupervised domain adaptation (IOSUDA)—to address performance degradation in joint OD and OC segmentation. Furthermore, reference [6] utilized a deep learning method based on M-Net, which applies a polar coordinate transformation to convert fundus images into a polar coordinate system. Subsequently, the transformed images are processed using M-Net, a multi-label deep network that generates a probability map containing OD and OC regions. To address the issue of automatic OD center localization and long training time, reference [7] proposed an approach utilizing the fully convolutional network FC-DenseNet. However, this method has its limitations. In reference [8], an improved fully convolutional network (FCN) was utilized for preprocessing and simultaneous segmentation of the OD and OC. Reference [9] presented a transferable attention U-Net (TAU) model for OD and OC segmentation across different fundus image datasets. This model incorporates two discriminators and attention modules, to localize and extract invariant features across datasets. Reference [10] introduced EDDense-Net, a segmentation network for estimating the cup-to-disc ratio (CDR) for glaucoma diagnosis. Using dense blocks and grouped convolutional layers, it captures spatial information and reduces complexity. Evaluated on two public datasets, EDDense-Net outperformed existing methods in accuracy and efficiency, aiding ophthalmologists in diagnosing glaucoma. Reference [11] proposed a modified U-Net model for detecting the edges of the optic cup and disc in fundus images of glaucoma patients. The approach utilized edge detection and dilation techniques to enhance segmentation. A novel boundary-enhanced adaptive context network known as BEAC-Net was introduced in reference [12], designed for accurate segmentation of both the optic disc and the optic cup in retinal fundus images. BEAC-Net integrates an efficient boundary pixel attention (EBPA) module and an adaptive context module (ACM), to improve boundary detection and capture contextual information. Reference [13] presented LC-MANet, a multiplex aggregation network for joint segmentation of the optic disc (OD) and optic cup (OC), to estimate the cup-to-disc ratio (CDR) for glaucoma diagnosis. It integrates independent and joint segmentation with a coarse-to-fine approach and uses multi-channel fusion to reduce interference. The method outperforms existing techniques in accuracy on the RIM-ONE, Drishti-GS, and REFUGE datasets.

Despite these advances, deep learning-based optic disc (OD) and optic cup (OC) segmentation still faces several challenges. Firstly, the structure of the segmentation models is usually complicated, which leads to high computation cost and long segmentation time. Maintaining a balance between segmentation accuracy and computational cost and time remains a challenge. Secondly, the model has limited generalization ability on different datasets, making it difficult to achieve consistent performance on diverse datasets. Finally, the model’s complexity and the substantial size of its weight files pose challenges for deployment and use on mobile devices with limited resources.

In this paper, we present an end-to-end network model for optic disc (OD) and optic cup (OC) segmentation that addresses existing challenges by minimizing computational demands and accelerating inference speed, thus enabling efficient deployment on mobile devices. Our methodology incorporates joint multi-label segmentation of the optic disc (OD) and optic cup (OC), complemented by an additional boundary branch aimed at improving segmentation accuracy. Furthermore, the use of adversarial learning techniques allows us to refine segmentation boundaries, further boosting accuracy. When compared to prior methods, our network model offers advantages including reduced parameter count, lower computational overhead, quicker inference, and improved segmentation accuracy.

The principal contributions of this paper are outlined as follows: (1) We present a comprehensive network for segmenting the optic disc and optic cup, designed to reduce computational overhead and enhance inference speed. By incorporating a lightweight feature-extraction network, we achieve improved segmentation efficiency while preserving accuracy. (2) Our method ensures a swift inference process, with a processing time of approximately 24 milliseconds, making it ideal for use in mobile devices and in clinical settings. (3) By leveraging multi-label segmentation and adversarial learning techniques, we refine boundary delineation, which boosts the segmentation accuracy for both the optic disc and the optic cup and enhances the model’s overall generalization performance.

## 2. Methods

### 2.1. Generating the Network

**Figure 1:**
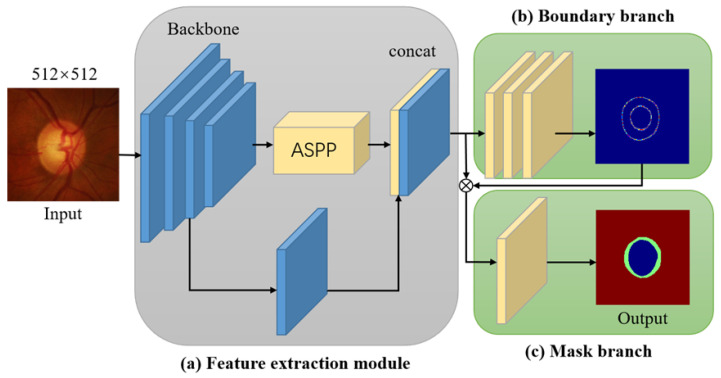
Overview of the MBG-Net network architecture: (**a**) is the feature-extraction module; (**b**) is the boundary branch; (**c**) is the mask branch.

The paper proposes a lightweight segmentation network that inherits the overall structure of Deeplabv3+ [14]. The architecture of the MBG-Net network is illustrated in Figure 1. After the feature-extraction network, it connects a spatial pyramid pooling module (Atrous Spatial Pyramid Pooling, ASPP) with atrous convolution. Meanwhile, it integrates more feature information by fusing the shallow features of the feature-extraction network with the feature map acquired from the spatial pyramid pooling module, with the aim of obtaining more comprehensive feature information. The difference is that the Deeplabv3+ network uses the ResNet [15] series as its feature-extraction network. ResNet networks have excellent performance in feature extraction but come with a large amount of computation, which is contrary to the goal of designing a lightweight and efficient segmentation network model. Inspired by successful applications of the MobileNet [16,17,18] series on mobile devices, this paper replaces ResNet with MobileNet as its feature-extraction network and proposes a feature-extraction method based on MobileNetv3.

In terms of specific operations, MBG-Net utilizes the large version of the MobileNetv3 network as its feature-extraction network. However, only the first convolutional layer and 15 inverted residual blocks from the large MobileNetv3 version are utilized. Additionally, the stride of the third-to-last inverted residual block is modified from 2 to 1. Experimental comparisons have demonstrated that this alteration is advantageous for extracting local features. To better extract contextual information, multi-scale feature fusion is performed on the extracted feature maps, using the spatial pyramid pooling module with atrous convolution. Subsequently, the feature map obtained from the spatial pyramid pooling module undergoes operations like batch normalization (BN) and rectified linear unit (ReLU) with a convolution kernel of size 1 × 1, to reduce the number of feature map channels. Afterwards, the feature map is upsampled fourfold, and the resulting feature map is combined with the feature map obtained from the third inverted residual block of MobileNetv3.

To enhance the accuracy of OD and OC segmentation, a multi-label segmentation method is used, where the resulting feature maps are input into the boundary prediction branch and the mask prediction branch. Specifically, for the boundary branch, the feature map undergoes three convolution operations, where the output channels of the first two convolutions are set to 256. The final convolution operation produces a feature map with a single channel, resulting in the predicted boundaries for both the OD and OC. For the mask branch, the feature map is concatenated with the predicted boundary feature map and then subjected to a convolution operation. The output feature map consists of two channels, which are in correspondence with the predicted masks for the optic disc (OD) and optic cup (OC). The obtained mask features are then upsampled by a factor of four, to generate a mask prediction map that matches the input image’s size. All the aforementioned convolution operations use a stride of one. To enhance the accuracy of boundary and mask predictions, the boundary regression loss and mask prediction loss are formulated as defined in Equations (1) and (2), respectively:

(1) $$L_{b}=\frac{1}{N}\sum_{i}^{N}{\left(y_{i}^{b}-p_{i}^{b}\right)}^{2}$$

(2) $$L_{m}=-\frac{1}{N}\sum_{i}^{N}\left[y_{i}^{m}*\log\left(p_{i}^{m}\right)+\left(1-y_{i}^{m}\right)\log\left(1-p_{i}^{m}\right)\right]$$

For Formula (1), *N* denotes the total number of pixels, $y_{i}^{b}$ is the natural boundary map generated by morphological closure operation and Gaussian filter, and $p_{i}^{b}$ is the boundary prediction map generated by the segmentation network. For Equation (2), $y_{i}^{m}$ is the ground-truth mask label and $p_{i}^{m}$ is the mask prediction map predicted by the segmentation network.

The method in this paper sends the mask prediction map to the patch discriminator (PatchGAN) to deceive the patch discriminator, in order to optimize the parameters of the entire segmentation network, allowing the network to generate a more realistic prediction map. The segmentation network is optimized using the subsequent adversarial loss:

(3) $$L_{adv}=-\frac{1}{N}\sum_{i}^{N}L_{bce}\left(p^{m},1\right)$$

Joint Loss function: We can consider loss function that integrates both the segmentation loss and the boundary loss as follows:

(4) $$L_{total}=L_{b}+L_{m}+\beta *L_{adv}$$

To summarize, the loss for the segmentation network comprises the boundary prediction loss, the mask prediction loss, and the adversarial loss. β is the balance coefficient, which is employed to balance the proportion of the adversarial loss function. Based on empirical evidence, β is set to 0.01.

### 2.2. Adversarial Networks

A PatchGAN is employed as the adversarial network for MBG-Net. The concept of adversarial learning aims to achieve optimal segmentation effects through a maximization and minimization game between the generator and the discriminator. After comparing discriminators, such as ImageGAN and PixelGAN, PatchGAN was chosen as the adversarial generative network for MBG-Net. PatchGAN can capture local information in the output space, allowing the segmentation network to emphasize local structural similarities within image patches. This adversarial approach ensures that the segmentation masks adhere to geometric constraints.

**Figure 2:**
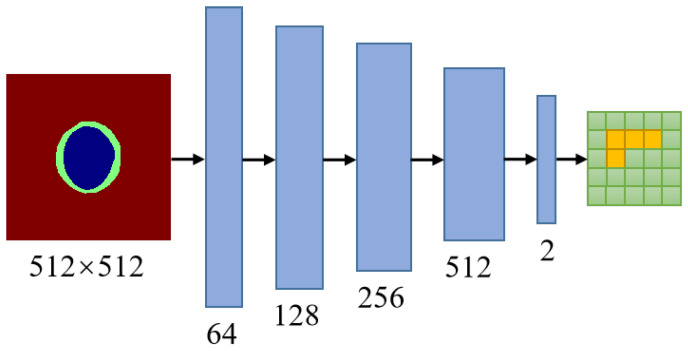
An overview of discriminator network architecture.

Concretely, a PatchGAN is connected after the mask branch. As illustrated in Figure 2, the PatchGAN network encompasses five convolutional layers. The size of the convolutional kernels is set to 4 × 4, with a stride of 2. The output channels of the five convolutional layers increase progressively from the shallower to the deeper layers, with magnitudes of 64, 128, 256, and 512, respectively. The final output channel is 2. The activation function following the last convolutional layer is the Sigmoid function, while for the other convolutional layers, the activation functions are LeakyReLU with a negative slope value of 0.2.

For the parameter training of the patch discriminator network, Equation (5) is used for optimization, to distinguish whether the mask comes from the segmentation network or the generator. An interactive training strategy between the generator and the discriminator is employed during the training process, to optimize the parameters of the entire network. A set of optimal model parameters is learned through the max–min game between the generator and the discriminator.

(5) $$L_{D}=-\frac{1}{N}\sum_{i}^{N}\left[L_{bce}\left(p^{m},0\right)+L_{bce}\left(y^{m},1\right)\right]$$

In Equation (5), $p^{m}$ denotes the mask prediction map, while $y^{m}$ signifies the manually annotated mask map.

## 3. Experiments and Results

### 3.1. Datasets

**Figure 3:**
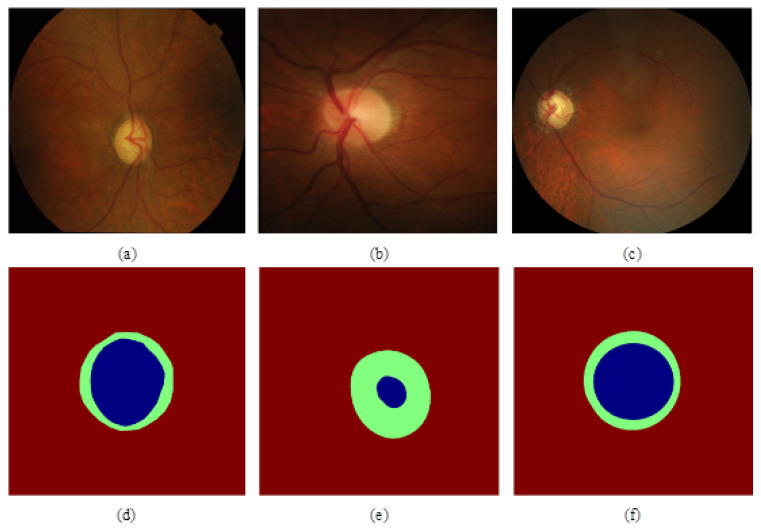
The sample images and corresponding annotation images of the Drishti-GS, RIM-ONE-r3, and REFUGE datasets are presented as follows: (**a**,**d**) represent the sample images and corresponding annotations of the Drishti-GS dataset; (**b**,**e**) are the RIM-ONE-r3 dataset sample map and the corresponding annotation map; (**c**,**f**) are the REFUGE dataset sample map and the corresponding annotation map.

The experiment utilized three publicly accessible fundus datasets: Drishti-GS, RIM-ONE-r3, and REFUGE. The Drishti-GS dataset consists of 101 fundus images, which include 31 normal and 70 glaucomatous images. The RIM-ONE-r3 dataset contains 159 fundus images, with 85 normal and 74 glaucomatous images. As the largest open-source glaucoma fundus dataset available to date, the REFUGE dataset contains 1200 fundus images divided into three subsets: 400 for training, 400 for validation, and another 400 for testing purposes. The sample graphs and annotation graphs of the three datasets are shown in Figure 3: (a) and (d) show the glaucoma fundus sample map alongside its annotation from the Drishti-GS dataset; (b) and (e) depict the normal eye fundus sample map and the corresponding annotation map from the RIM-ONE-r3 dataset; while (c) and (f) depict both the glaucoma fundus sample map and its annotation from REFUGE. In (d), (e), and (f), green indicates the optic disc, whereas blue denotes the optic cup. The experiment used 320 images from the REFUGE dataset for training and validation and 80 images for testing. The specific data of the three datasets are shown in Table 1:

### 3.2. Implementation Details

The experimental model was built using the PyTorch deep learning framework. At first, the segmentation network was trained, and, subsequently, an adversarial learning network was introduced, to optimize the parameters of the entire MBG-Net network. A set of optimal model parameters was obtained through alternate training. The Adam optimizer was adopted for training the segmentation network, and the stochastic gradient descent (SGD) algorithm was utilized when training the patch discriminator network. In the experiment, the pre-trained weights on the ImageNet dataset were used as the initialization weights of the feature-extraction network, namely, MobileNetv3_Large. The initial learning rates of the segmentation network and the patch discriminator were set to $1\times 10^{-3}$ and $2.5\times 10^{-5}$, respectively, and Momentum was set to 0.9. The experiments were trained on an NVIDIA GTX 1080Ti GPU (NVIDIA Corporation, Santa Clara, CA, USA) with a batch size of 8 for 300 epochs. For the region of interest (ROI) extraction, we followed the method of [20]. First, we used the U-Net network for rough OD localization, then a 512×512 area centered on the OD mask was cropped, as input for MBG-Net. Due to the limited availability of fundus images, which makes public datasets relatively small, we employed several data augmentation techniques, to enhance the quantity and diversity of the images. These techniques included random scaling, rotation, flipping, elastic transformation, contrast adjustment, noise addition, and random erasing. Additionally, to further refine the segmentation results, we applied morphological operations and median filtering to the prediction masks. This process involved techniques such as hole filling and selecting the largest connected region, to achieve smoother and more natural boundaries.

### 3.3. Algorithm Evaluation

The Dice index (DI) is a standard evaluation index for the segmentation task, and the CDR is one of the crucial indicators for clinical glaucoma screening. This paper adopted DI and CDR to evaluate the segmentation performance of MBG-Net. The evaluation criteria were defined as follows:

(6) $$Dice=\frac{\left(2\times N\_TP\right)}{\left((2\times N\_TP)+N\_FP+N\_FN\right)}$$

(7) $$CDR=\frac{VCD}{VDD}$$

(8) $$\delta_{cdr}=\sum_{i=1}^{N}\left|CDR_{i}^{S}-CDR_{i}^{G}\right|$$

N_TP,N_FP,andN_FN denoted the number of pixels corresponding to true positives, false positives, and false negatives, respectively. VCD and VDD represented the vertical diameters of the cup and the disc, respectively, which were calculated from the segmentation results of the cup and the disc. CDR represented the ratio of VCD and VDD. $CDR^{G}$ and $CDR^{S}$ represented the vertical cup-to-disc ratio obtained from the true values and the predicted segmentation, respectively. *N* represented the number of test samples. The $\delta_{cdr}$ was defined as a measure of the precision of the CDR estimate, which calculated the average error rate for all the samples, as shown in Equation (8). Lower values of $\delta_{cdr}$ signified better prediction results.

### 3.4. Experimental Results

**Figure 4:**
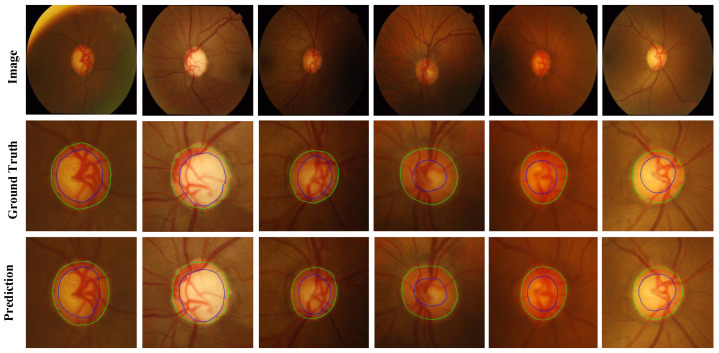
Prediction results of the Drishti-GS dataset (Image is the fundus picture, Ground Truth is the annotation map, and Prediction is the network prediction map).

**Figure 5:**
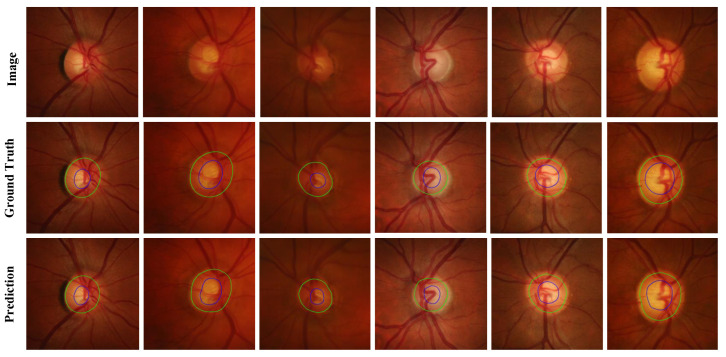
Prediction results of the RIM-ONE-r3 dataset (Image is the fundus picture, Ground Truth is the label map, and Prediction is the network prediction map).

For the Drishti-GS and RIM-ONE-r3 datasets, we evaluated our MBG-Net against several leading OD and OC segmentation methods: pOSAL [20], BEAL [22], TD-GAN [23], BGA-net [24], Sevastopolsky [25], Zilly [26], BEAC-Net [12], LC-MANet [13], etc. The quantitative results are presented in Table 2, while the qualitative results are illustrated in Figure 4 and Figure 5.

**Figure 6:**
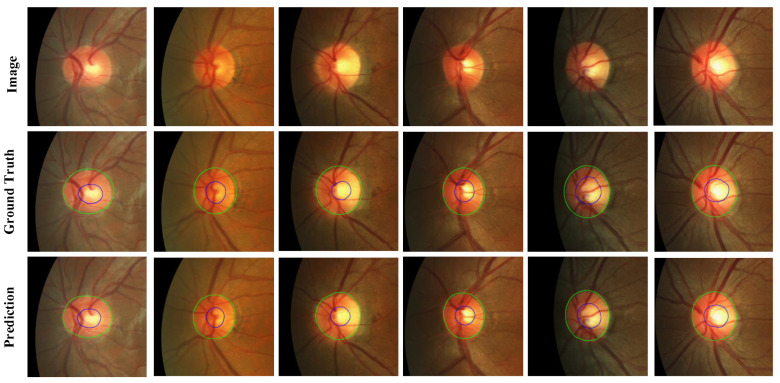
Prediction results of the REFUGE-train dataset (Image is the fundus picture, Ground Truth is the annotation map, and Prediction is the network prediction map).

For the REFUGE dataset, its training set was used as experimental data. We took 320 images as the training set and 80 as the test set. Compared to the BGA-net segmentation model, which shows better performance in Table 3, the network model proposed in this paper improved the inference time by about 16% and also enhanced the segmentation accuracy of the OC. Additionally, the index also showed specific improvement. The quantitative results are shown in Table 3, and Figure 6 shows the qualitative results of the REFUGE dataset.

Additionally, this paper compared the model parameters, memory usage, computational cost, and inference time of the network models with different feature-extraction networks, to demonstrate the excellent performance and lightweight nature of MobileNetv3 as a feature-extraction network. The specific results are shown in Table 4.

**Figure 7:**
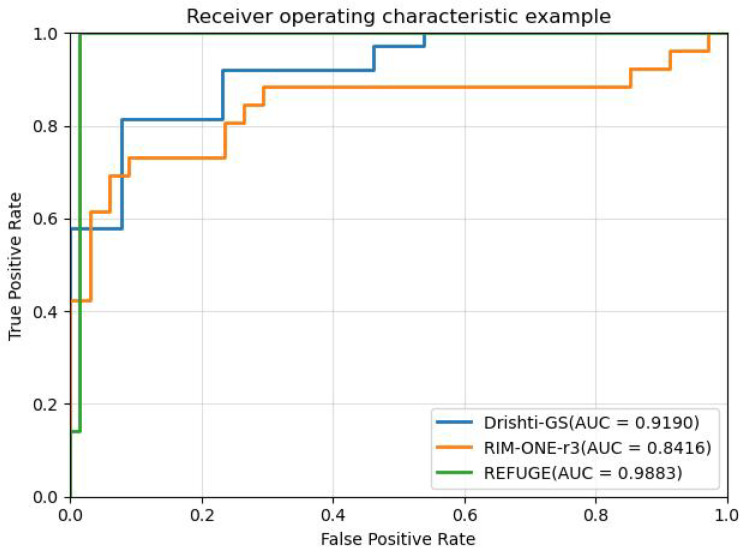
ROC curve and AUC evaluation results of different datasets on BGA-Net.

The cup-to-disc ratio (CDR) is a crucial clinical parameter for diagnosing glaucoma, and it serves as a fundamental basis for diagnosis by most ophthalmologists. In this paper, the segmentation performance of BGA was evaluated by means of the receiver operating characteristic (ROC) curve and the corresponding area under the curve (AUC). Generally speaking, a higher AUC implies superior diagnostic performance, and greater accuracy reflects the enhancement of algorithm performance. Figure 7 presents the ROC curves and the corresponding AUC values on three public datasets, namely, Drishti-GS, RIM-ONE-r3, and REFUGE-train.

### 3.5. Ablation Experiments

The subsequent ablation experiments aimed to assess the effectiveness of both the boundary branch and the discriminator network: (1) baseline (feature-extraction network + ASPP + mask branch); (2) baseline + boundary branch; (3) baseline + boundary branch + discriminator network.

**Figure 8:**
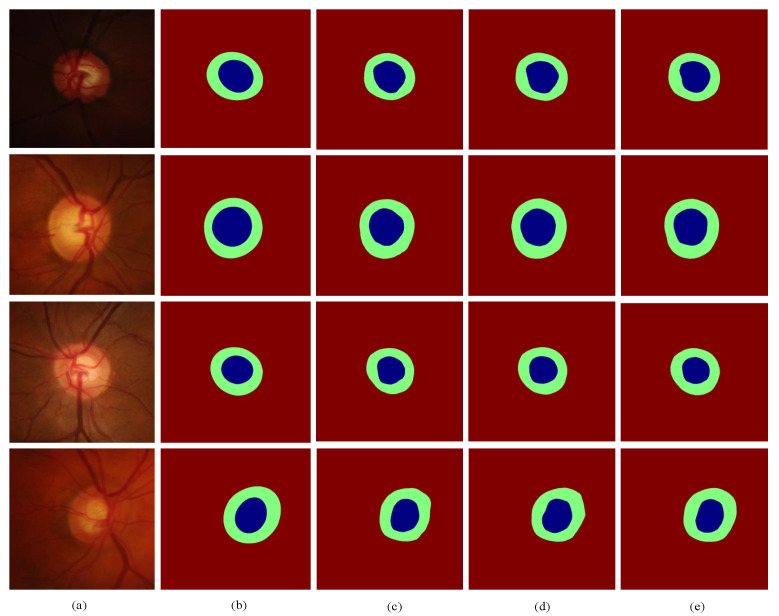
Comparison of qualitative segmentation results under different module combinations: (**a**) the ROI area of the fundus image; (**b**) the manual annotation map; (**c**) the predicted segmentation map associated with the baseline model; (**d**) the predicted segmentation map for baseline + boundary branch; and (**e**) the predicted segmentation map for baseline + boundary branch + discriminator network (MBG-Net).

Ablation experiments were performed on the RIM-ONE-r3 dataset, and the experimental results of the different module combinations are shown in Table 5. The experiments demonstrated that, in comparison to the baseline network, both the boundary branch and the discriminator network significantly enhanced the model’s segmentation performance, to varying degrees. The network structure with boundary branches added to the baseline improved the OC segmentation accuracy by 0.3% and decreased the mean absolute error ($\delta_{cdr}$) by 0.004. Furthermore, when both the discriminator and the boundary branch components were incorporated, the network structure of MBG-Net enhanced the OC accuracy by 0.7% compared to the baseline with boundary branch, and reduced the mean absolute error ($\delta_{cdr}$) by 0.003, which proves the effectiveness of the boundary branch and the discriminator network. Meanwhile, the qualitative segmentation results are given in Figure 8: green indicates the optic disc (OD); blue represents the optic cup (OC); column (a) is the ROI area corresponding to the fundus image; column (b) is the manual annotation map; column (c) is the predicted segmentation map corresponding to the baseline; column (d) is the predicted segmentation map corresponding to baseline + boundary branch; and column (e) is the predicted segmentation map corresponding to baseline + boundary branch + discriminator network (MBG-Net). Compared with columns (c) and (d), the boundary of the segmentation map in column (e) is smoother and more natural, and it is closer to the manual annotation map.

Furthermore, to verify the effectiveness of data preprocessing, we compared the original images and the experimental images with ROI cropping on the Refuge dataset, as shown in Table 6. The cropped dataset was able to effectively improve the OD and OC Dice.

## 4. Discussion

The CDR is an essential attribute in diagnosing glaucoma, and accurate segmentation of the OD and OC is crucial to the precise acquisition of the CDR. In recent years, OD and OC segmentation methods based on deep learning have made significant progress. However, there is still a significant gap between research work and clinical application. Most of the current segmentation networks have a large number of model parameters and a long segmentation time, which cannot meet the clinical needs of mobile deployment and real-time detection. This paper proposes a lightweight MBG-Net segmentation model for the above problems. Our experiments show that the proposed method has reached an advanced level, in terms of segmentation accuracy, computational cost, model parameters, etc., indicating the application potential of the method in mobile deployment and real-time detection.

Our experiments performed on the Drishti-GS, RIM-ONE-r3, and REFUGE datasets demonstrated that the proposed method yielded the most advanced segmentation results and the lowest absolute error $\delta_{cdr}$ with the least amount of parameters. Using the ROC curve to evaluate the model’s performance, the accuracy rates on the three datasets were 91.90%, 84.16%, and 98.83%, respectively. In the experiment, the ROI region was extracted from the original image according to the method in the literature, and the extracted ROI region was used as the input of MBG-Net. To prove the necessity of ROI region extraction, the following experiments were carried out on MBG-Net: keeping the original experimental conditions unchanged, the original dataset REFUGE-train was trained and tested on MBG-Net, and the results are shown in Table 3, which shows that the segmentation accuracy of the OD decreased by 4.2% and that the segmentation accuracy of the OC decreased by 6%. This experiment shows the effectiveness and necessity of ROI extraction. In addition, the inference time of this network for an image on a single NVIDIA GTX 1080Ti GPU was only 24.3 ms, which shows the high efficiency and real-time performance of the MBG-Net network.

To show the segmentation effect intuitively, this paper presents part of the segmentation renderings of the three datasets. The comparison shows that the segmentation effects of this method on the three datasets are comparable to those of the manual annotation images, which illustrates the generalization capability of this method.

## 5. Conclusions

This paper presents MBG-Net, an optimized network specifically developed for the segmentation of the optic disc (OD) and optic cup (OC). MBG-Net specifically addresses the challenges associated with OD and OC segmentation by integrating these tasks into a unified framework. The network is optimized to deliver reduced training and computational expenses, quicker inference times, and enhanced segmentation accuracy. It leverages MobileNetv3 for lightweight feature extraction and incorporates the boundary auxiliary branches alongside adversarial learning techniques, to boost segmentation performance while maintaining accuracy. We validated the effectiveness of MBG-Net through extensive experiments on three widely used fundus datasets. Our results demonstrate that the network not only achieves superior segmentation performance but also maintains low $\delta_{cdr}$ values across all datasets, underscoring its efficacy in tackling OD and OC segmentation challenges and supporting glaucoma diagnosis. In the future, we will produce a further paper on how to deploy web applications to the network for mobile terminals to achieve real-time glaucoma-assisted detection.

## Figures and Tables

**Table 1:** Statistics of the datasets used in evaluating our method.

Dataset	Number of Samples	Image Size	Cameras	Release Year
Drishti-GS [19]	50 + 51	2047 × 1759	unknown	2014
RIM-ONE-r3 [20]	99 + 60	2144 × 1424	unknown	2015
REFUGE [21]	320 + 80	2124 × 2056	Zeiss Visucam 500	2018

**Table 2:** Performance comparison of attention blocks for optic disc and optic cup segmentation on Drishti-GS and Rim_One_r3 datasets (mIoU was used to select the best model).

Methods	Datasets					
	Drishti-GS			Rim_One_r3		
	${\mathrm{DI}}_{\mathrm{disc}}$	${\mathrm{DI}}_{\mathrm{cup}}$	$\delta_{\mathrm{cdr}}$	${\mathrm{DI}}_{\mathrm{disc}}$	${\mathrm{DI}}_{\mathrm{cup}}$	$\delta_{\mathrm{cdr}}$
U-net [19]	0.904	0.852	-	0.864	0.797	-
M_UNet [25]	0.95	0.85	-	0.95	0.82	-
M-Net [6]	0.967	0.808	-	0.952	0.802	-
CE-Net [27]	0.964	0.882	-	0.953	0.844	-
CDED-Net [28]	0.959	0.924	-	0.958	0.862	-
pOSAL [20]	0.965	0.858	0.082	0.865	0.787	0.081
Gan-based [23]	0.953	0.864	-	0.953	0.825	-
BEAL [22]	0.961	0.862	-	0.898	0.810	-
PDD-UNET [29]	0.963	0.848	0.105	0.970	0.876	0.066
BEAC-Net [12]	0.8614	0.8087	-	0.8582	0.7333	-
LC-MANet [13]	0.9723	0.9034	0.043	0.9729	0.8458	0.0444
Ours	0.974	0.900	0.045	0.966	0.875	0.043

**Table 3:** Performance comparison of Dice coefficient of OD and OC segmentation on REFUGE [30] dataset.

Methods	$D_{\mathrm{disc}}$	$D_{\mathrm{cup}}$	$\delta_{\mathrm{cdr}}$	Inference Time
U-net [19]	0.927	0.848	-	-
pOSAL [20]	0.932	0.869	0.059	-
Psi-Net [21]	0.956	0.851	-	-
BEAL [22]	0.945	0.860	-	-
BGA-NET [24]	0.951	0.866	0.040	29.1 ms
ours	0.951	0.869	0.013	24.3 ms

**Table 4:** Performance comparison of network models under different feature-extraction networks.

Backbone	Total Params	Total Memory	Total MAdd	Total Flops	Total MemR + W	Segmentation Time
efficientnetv2_m	54.9 M	2299.8 M	158.4 G	79.3 G	3.1 GB	192.7 ms
Xception	52.1 M	1524.5 M	165.8 G	83.0 G	3.1 GB	170.4 ms
Resnet50	38.4 M	845.3 M	138.5 G	69.3 G	1.7 GB	58.9 ms
Mobilenetv2	5.5 M	651.1 M	52.9 G	26.5 G	1.2 GB	29.1 ms
Ours	5.3 M	495.4 M	48.8 G	24.4 G	839.0 M	24.3 ms

**Table 5:** Ablation paper on different components.

Dataset	${\mathrm{DI}}_{\mathrm{disc}}$	${\mathrm{DI}}_{\mathrm{cup}}$	$\delta_{\mathrm{cdr}}$
Baseline	0.964	0.865	0.050
Baseline + B	0.964	0.868	0.046
Baseline + B + G	0.966	0.875	0.043

**Table 6:** The necessity of ROI region extraction on REFUGE [30] dataset.

Dataset	$D_{\mathrm{disc}}$	$D_{\mathrm{cup}}$
Original dataset	0.920	0.820
ROI extraction dataset	0.962	0.880

## Data Availability

All data are available in the public domain.

## References

[1] Tham Y.C., Li X., Wong T.Y., Quigley H.A., Aung T., Cheng C.Y. (2014). Global prevalence of glaucoma and projections of glaucoma burden through 2040: A systematic review and meta-analysis. Ophthalmology.

[2] Mary V.S., Rajsingh E.B., Naik G.R. (2016). Retinal fundus image analysis for diagnosis of glaucoma: A comprehensive survey. IEEE Access.

[3] Chen N., Lv X. (2024). Research on segmentation model of optic disc and optic cup in fundus. BMC Ophthalmol..

[4] Zhu Q., Chen X., Meng Q., Song J., Luo G., Wang M., Shi F., Chen Z., Xiang D., Pan L. (2021). GDCSeg-Net: General optic disc and cup segmentation network for multi-device fundus images. Biomed. Opt. Express.

[5] Chen C., Wang G. (2021). IOSUDA: An unsupervised domain adaptation with input and output space alignment for joint optic disc and cup segmentation. Appl. Intell..

[6] Fu H., Cheng J., Xu Y., Wong D.W.K., Liu J., Cao X. (2018). Joint optic disc and cup segmentation based on multi-label deep network and polar transformation. IEEE Trans. Med. Imaging.

[7] Al-Bander B., Williams B.M., Al-Nuaimy W., Al-Taee M.A., Pratt H., Zheng Y. (2018). Dense fully convolutional segmentation of the optic disc and cup in colour fundus for glaucoma diagnosis. Symmetry.

[8] Qin P., Wang L., Lv H. (2019). Optic disc and cup segmentation based on deep learning. Proceedings of the 2019 IEEE 3rd Information Technology, Networking, Electronic and Automation Control Conference (ITNEC).

[9] Zhang Y., Cai X., Zhang Y., Kang H., Ji X., Yuan X. (2021). TAU: Transferable Attention U-Net for optic disc and cup segmentation. Knowl.-Based Syst..

[10] Mehmood M., Naveed K., Khan H.A., Naqvi S.S. (2023). EDDense-Net: Fully Dense Encoder Decoder Network for Joint Segmentation of Optic Cup and Disc. arXiv.

[11] Tadisetty S., Chodavarapu R., Jin R., Clements R.J., Yu M. (2023). Identifying the edges of the optic cup and the optic disc in glaucoma patients by segmentation. Sensors.

[12] Jiang L., Tang X., You S., Liu S., Ji Y. (2023). BEAC-Net: Boundary-Enhanced Adaptive Context Network for Optic Disk and Optic Cup Segmentation. Appl. Sci..

[13] Yu J., Chen N., Li J., Xue L., Chen R., Yang C., Xue L., Li Z., Wei L. (2024). LC-MANet: Location-constrained joint optic disc and cup segmentation via multiplex aggregation network. Comput. Electr. Eng..

[14] Chen L.C., Zhu Y., Papandreou G., Schroff F., Adam H. Encoder-decoder with atrous separable convolution for semantic image segmentation. Proceedings of the European Conference on Computer Vision (ECCV).

[15] He K., Zhang X., Ren S., Sun J. Deep residual learning for image recognition. Proceedings of the IEEE Conference on Computer Vision and Pattern Recognition.

[16] Howard A.G., Zhu M., Chen B., Kalenichenko D., Wang W., Weyand T., Andreetto M., Adam H. (2017). Mobilenets: Efficient convolutional neural networks for mobile vision applications. arXiv.

[17] Sandler M., Howard A., Zhu M., Zhmoginov A., Chen L.C. Mobilenetv2: Inverted residuals and linear bottlenecks. Proceedings of the IEEE Conference on Computer Vision and Pattern Recognition.

[18] Howard A., Sandler M., Chu G., Chen L.C., Chen B., Tan M., Wang W., Zhu Y., Pang R., Vasudevan V. Searching for mobilenetv3. Proceedings of the IEEE/CVF International Conference on Computer Vision.

[19] Ronneberger O., Fischer P., Brox T. (2015). U-net: Convolutional networks for biomedical image segmentation. Proceedings of the International Conference on Medical Image Computing and Computer-Assisted Intervention.

[20] Wang S., Yu L., Yang X., Fu C.W., Heng P.A. (2019). Patch-based output space adversarial learning for joint optic disc and cup segmentation. IEEE Trans. Med. Imaging.

[21] Murugesan B., Sarveswaran K., Shankaranarayana S.M., Ram K., Joseph J., Sivaprakasam M. (2019). Psi-Net: Shape and boundary aware joint multi-task deep network for medical image segmentation. Proceedings of the 2019 41st Annual International Conference of the IEEE Engineering in Medicine and Biology Society (EMBC).

[22] Wang S., Yu L., Li K., Yang X., Fu C.W., Heng P.A. (2019). Boundary and entropy-driven adversarial learning for fundus image segmentation. Proceedings of the Medical Image Computing and Computer Assisted Intervention–MICCAI 2019: 22nd International Conference.

[23] Son J., Park S.J., Jung K.H. (2019). Towards accurate segmentation of retinal vessels and the optic disc in fundoscopic images with generative adversarial networks. J. Digit. Imaging.

[24] Luo L., Xue D., Pan F., Feng X. (2021). Joint optic disc and optic cup segmentation based on boundary prior and adversarial learning. Int. J. Comput. Assist. Radiol. Surg..

[25] Sevastopolsky A. (2017). Optic disc and cup segmentation methods for glaucoma detection with modification of U-Net convolutional neural network. Pattern Recognit. Image Anal..

[26] Zilly J., Buhmann J.M., Mahapatra D. (2017). Glaucoma detection using entropy sampling and ensemble learning for automatic optic cup and disc segmentation. Comput. Med. Imaging Graph..

[27] Gu Z., Cheng J., Fu H., Zhou K., Hao H., Zhao Y., Zhang T., Gao S., Liu J. (2019). Ce-net: Context encoder network for 2d medical image segmentation. IEEE Trans. Med. Imaging.

[28] Tabassum M., Khan T.M., Arsalan M., Naqvi S.S., Ahmed M., Madni H.A., Mirza J. (2020). CDED-Net: Joint segmentation of optic disc and optic cup for glaucoma screening. IEEE Access.

[29] Shankaranarayana S.M., Ram K., Mitra K., Sivaprakasam M. (2019). Fully convolutional networks for monocular retinal depth estimation and optic disc-cup segmentation. IEEE J. Biomed. Health Inform..

[30] Orlando J.I., Fu H., Breda J.B., Keer K.V., Bathula D.R., Diaz-Pinto A., Fang R., Heng P.A., Kim J., Lee J.H. (2019). REFUGE Challenge: A Unified Framework for Evaluating Automated Methods for Glaucoma Assessment from Fundus Photographs. Med. Image Anal..

